# Synergistic anti-tumor activity of ciclopirox olamine and metformin in triple-negative breast cancer

**DOI:** 10.1016/j.gendis.2025.101538

**Published:** 2025-01-22

**Authors:** Yakun Wu, Hui Lyu, Congcong Tan, Margaret E. Larsen, Shou-Ching Tang, Shile Huang, Bolin Liu

**Affiliations:** a Departments of Interdisciplinary Oncology and Genetics, LSU LCMC Health Cancer Center, School of Medicine, Louisiana State University Health Sciences Center, New Orleans, Louisiana 70112, USA; b Department of Medicine, LSU LCMC Health Cancer Center, School of Medicine, Louisiana State University Health Sciences Center, New Orleans, Louisiana 70112, USA; c Department of Biochemistry and Molecular Biology, Louisiana State University Health Sciences Center, Shreveport, Louisiana 71103, USA

Triple-negative breast cancer (TNBC) represents a challenging subtype of breast cancer. High heterogeneity and lack of effective targeted therapies leave TNBC treatment a big challenge.^1^ However, repurposing existing drugs clinically proven to be safe and have low or no side effects is a potential choice.^2^ Recent research has also highlighted the potential benefits of combining pharmacological agents to enhance therapeutic outcomes.^3^ Studies show that ciclopirox olamine (CPX), an off-patent anti-fungal drug, and metformin (Met), a safe and first-line drug for treating type II diabetes, both can activate the apoptotic pathways. However, whether the combinations would exert potent anti-tumor activity against TNBC remains unclear. Here, we investigated the combinatorial anti-tumor effects of CPX and Met on TNBC. Our studies uncovered a synergistic impact of CPX in combination with Met, which resulted in a more profound inhibition of TNBC cell proliferation and a significantly enhanced apoptosis. The combinations activate both intrinsic and extrinsic apoptotic pathways in a caspase-dependent manner. Moreover, in a TNBC-derived tumor xenograft model, the combination, as compared with a single agent, potently suppressed tumor growth. Collectively, we demonstrate that the combinations of CPX and Met exhibit synergistic anti-tumor activity against TNBC *in vitro* and *in vivo*.

Previously, it was reported that CPX possessed anti-cancer activity in various types of human cancers.^4^ Alongside CPX, Met exhibits unique effects in inducing cell cycle S-phase arrest and apoptosis in TNBC but not in other subtypes of breast cancer cells.^5^ By comparing the anti-proliferative/anti-survival effects of CPX and Met on TNBC (HCC1806, MDA-MB-231, Hs578T, and MDA-MB-468) cells, we found that either CPX or Met inhibited the proliferation and survival of all the TNBC cells in a dose-dependent manner. However, different TNBC cell lines showed distinct sensitivity to the drug treatments (Fig. 1; Fig. S1). These data highlight the potential for optimizing drug dosages to achieve maximal efficacy via combinatorial treatments. To explore this hypothesis, we further performed MTS assays. Using ratios shown in Figure 1B and Figure S2, the combinations of CPX and Met, as compared with either agent alone, exhibited a much more potent activity to induce anti-proliferative/anti-survival effects in all cell lines tested. Indeed, the combination index calculated with the Chou-Talalay method was mainly less than 1, suggesting synergy between CPX and Met in the concentrations we used for the TNBC cell lines (Fig. 1B; Fig. S2).

**Figure 1:**
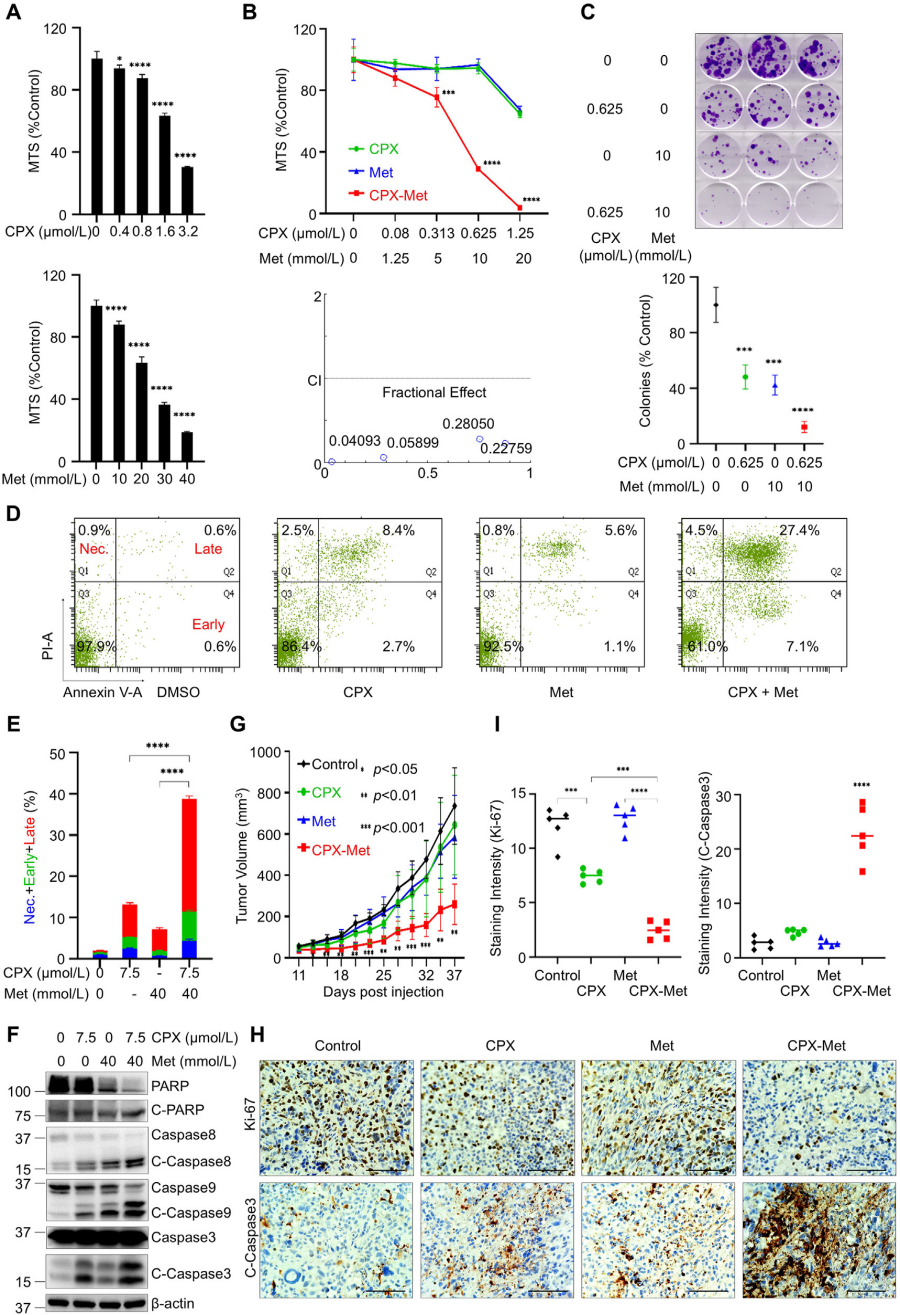
Combined anti-tumor activity of ciclopirox olamine (CPX) and metformin (Met) in triple-negative breast cancer. **(A)** Both CPX and Met inhibited the proliferation of HCC1806 cells in a dose-dependent manner. **(B)** Combinations of CPX and Met exhibited synergistic effects that inhibited the proliferation and survival of HCC1806 cells (upper panel); the combination index (bottom panel) of CPX and Met in treating HCC1806 cells. **(C)** CPX, in combination with Met, markedly suppressed the colony formation of HCC1806 cells. The relevant quantification of the number of colonies (bottom panel) was performed by calculating the percentages of colony numbers from each cell line relative to controls, defined as 100%, measured by Image J. CPX combined with Met profoundly induced cell death in HCC1806 cells. HCC1806 cells were cultured in 6 cm dishes with a medium containing 10% fetal bovine serum. After 24 h, cells were treated with indicated concentrations of CPX, Met, or CPX plus Met in a fresh medium containing 5% fetal bovine serum for another 48 h. All cells were collected and analyzed by flow cytometry; representative data were shown in **(D)**. The relevant quantification analysis was performed by calculating the percentages of the necrosis (Nec.), early apoptosis (Early), and late apoptosis (Late) population of cells from three independent replicates of the experiments **(E)**. CPX and Met together enhanced PARP cleavage and activation of caspases in HCC1806 cells. HCC1806 cells were treated with indicated concentrations of CPX, Met, or CPX plus Met in a fresh medium containing 5% fetal bovine serum and incubated for 48 h. All cells were collected, and the cell lysates were used for Western blot analyses with specific antibodies against PARP, caspase-8, caspase-9, caspase-3, and β-actin, as indicated **(F)**. Combinations of CPX and Met enhanced *in vivo* anti-tumor effects. **(G)** Tumor growth curves were plotted using average tumor volume within each group at the indicated time points. **(H)** At the end of treatment, tumor-bearing mice from the control and drug-treated groups were sacrificed. The tumors were dissected, and formalin-fixed paraffin-embedded sections of xenograft tumors were analyzed with immunohistochemistry staining for Ki67 and cleaved caspase-3 (C-Caspase 3) (scale bar, 100 μm). Quantifications of immunohistochemistry staining with ImageJ were shown in **(I)**. ∗*P* < 0.05, ∗∗*P* < 0.01, ∗∗∗*P* < 0.001, ∗∗∗∗*P* < 0.0001; bars: standard deviation.

Given that short-term combinations of CPX and Met showed synergistic effects, we next examined the long-term impact of the two drugs on TNBC cell colony formation. As shown in Figure 1C, when the combinatorial strategy was used repeatedly over a long period, profound inhibitory effects on TNBC cells were evident. In HCC1806 cells, CPX (0.625 μmol/L) in combination with Met (10 mmol/L) showed approximately 70% growth inhibition in the short-term MTS assays (Fig. 1B). In contrast, the combinations of the two drugs with the same doses displayed over 90% inhibition in the long-term colony formation assays (Fig. 1C). Moreover, the combinatorial effects of the two drugs on MDA-MB-231 cells observed in the short-term experiments were substantially amplified in the long-term assays. CPX (0.16 μmol/L) in combination with Met (2.5 mmol/L) slightly inhibited MDA-MB-231 cell growth in MTS assays (Fig. S2). However, their combinations dramatically suppressed (over 80%) colony formation of MDA-MB-231 cells (Fig. S3). The amplification effects were also observed in Hs578T and MDA-MB-468 cells (Fig. S3). Collectively, our data emphasizes the importance of long-term repeated combinatorial treatments for TNBC.

Previous data indicated that both CPX and Met, through different mechanisms, induced apoptosis in TNBC cells.^4^^,^^5^ Thus, it is conceivable to hypothesize that the combinations of CPX and Met may profoundly induce apoptosis in TNBC cells. To test this hypothesis, we performed flow cytometry analyses to detect early and late apoptosis and necrosis of TNBC cells. Treatment of HCC1806 cells with CPX or Met alone induced increased apoptosis, especially late apoptosis (Fig. 1D). The combinations of CPX and Met, as compared with either agent alone, stimulated more intensely and significantly increased both early and late apoptosis (Fig. 1D, E). Similar but not identical phenomena were also observed in other TNBC cells. In MDA-MB-231 and Hs578T cells, CPX, in combination with Met, as compared with either agent alone, induced much more necrosis and late apoptosis (Fig. S4A–D). However, treating BT549 cells by combining the two agents dramatically increased early and late apoptosis (Fig. S4E, F). Next, we conducted Western blot assays to investigate the potential mechanisms underlying the enhanced apoptotic effects. In HCC1806, MDA-MB-231, and BT549 cells, as compared with the single agent treatment, the combinations of CPX and Met led to an increased cleaved PARP, caspase-8, -9, and -3, suggesting activation of both the intrinsic and extrinsic apoptotic pathways (Fig. 1F; Fig. S5A). However, in Hs578T and MDA-MB-468 cells, our Western blot assays did not show higher cleaved PARP, caspase-8, -9, and -3 in the combined treatment groups than in the single treatment group. The cleavage levels of some of those apoptotic markers were even reduced to a certain extent (Fig. S5B). This could be due to more necrosis observed in Hs578T cells (Fig. S4C, D) and MDA-MB-468 cells (data not shown) upon CPX and Met treatment. Above all, these data suggest that combinations of CPX and Met induce activation of multiple caspases to trigger intrinsic and extrinsic apoptotic pathways in TNBC cells. However, necrosis may also occur in some TNBC cells. Our findings not only confirmed the high heterogeneity of TNBC cells and the diversity of their responses to drug treatments but also revealed the existence of other potential mechanisms involved in the combinations of CPX and Met.

To determine the *in vivo* anti-tumor activity of CPX and Met against TNBC, we established tumor xenograft models via injection of HCC1806 cells into the mammary fat pad of 6-week-old female nude mice. When tumors reached ∼60 mm^3^, the tumor-bearing mice started to be treated as indicated and then continued for the duration. By monitoring the progression of tumor growth, we found that the tumors in the combination-treated mice grew significantly slower than those in the control or single-agent-treated mice (Fig. 1G). The combinations also led to a substantially lower tumor weight and much smaller tumor size at the end of the experiment (Fig. S6A, B). There was no difference in the mouse body weight among the treatment groups (Fig. S6C), suggesting the dosage we used had little side effect. Next, we examined whether CPX and Met treatment would elicit anti-proliferation/anti-survival effects *in vivo*. To this end, we collected tumors at the end of animal experiments and performed immunohistochemistry analysis of Ki67, a typical cell proliferation marker, and cleaved caspase-3, an indicative of apoptosis. We discovered that treatment with the combinations of CPX and Met, as compared with the single agent, significantly decreased the expression of Ki67 and increased the tumor cells with positive staining for cleaved caspase-3 (Fig. 1H, I). Collectively, our data indicated that CPX, in combination with Met, exerted potent *in vivo* anti-tumor activity against TNBC and was associated with a significant inhibition of cell growth and induction of apoptosis. Given the challenging nature of TNBC treatment, our findings are promising as they suggest a potential new therapeutic strategy that utilizes existing drugs with well-characterized safety profiles.

## Ethics declaration

This study was approved by the Institutional Animal Care and Use Committee (IACUC) of Louisiana State University Health Sciences Center - New Orleans (LSUHSC-NO) (Protocol: 4833).

## Funding

This work was supported in part by a US National Institutes of Health/National Cancer Institute grant (No. R01CA266269) and a 10.13039/100008294Louisiana State University (LSU) Collaborative Cancer Research Initiative (CCRI) award (to B.L.).

## CRediT authorship contribution statement

**Yakun Wu:** Writing – review & editing, Writing – original draft, Methodology, Formal analysis, Data curation. **Hui Lyu:** Writing – review & editing, Methodology. **Congcong Tan:** Writing – review & editing, Methodology. **Margaret E. Larsen:** Writing – review & editing. **Shou-Ching Tang:** Writing – review & editing, Supervision. **Shile Huang:** Writing – review & editing, Funding acquisition, Conceptualization. **Bolin Liu:** Writing – review & editing, Writing – original draft, Supervision, Funding acquisition, Conceptualization.

## Conflicts of interests

Bolin Liu is an Editorial Board member of *Genes & Diseases*. He/she has no involvement in the peer-review of this article and has no access to information regarding its peer-review. The authors declared no conflict of interests.

## Figures and Tables

**figs1:**
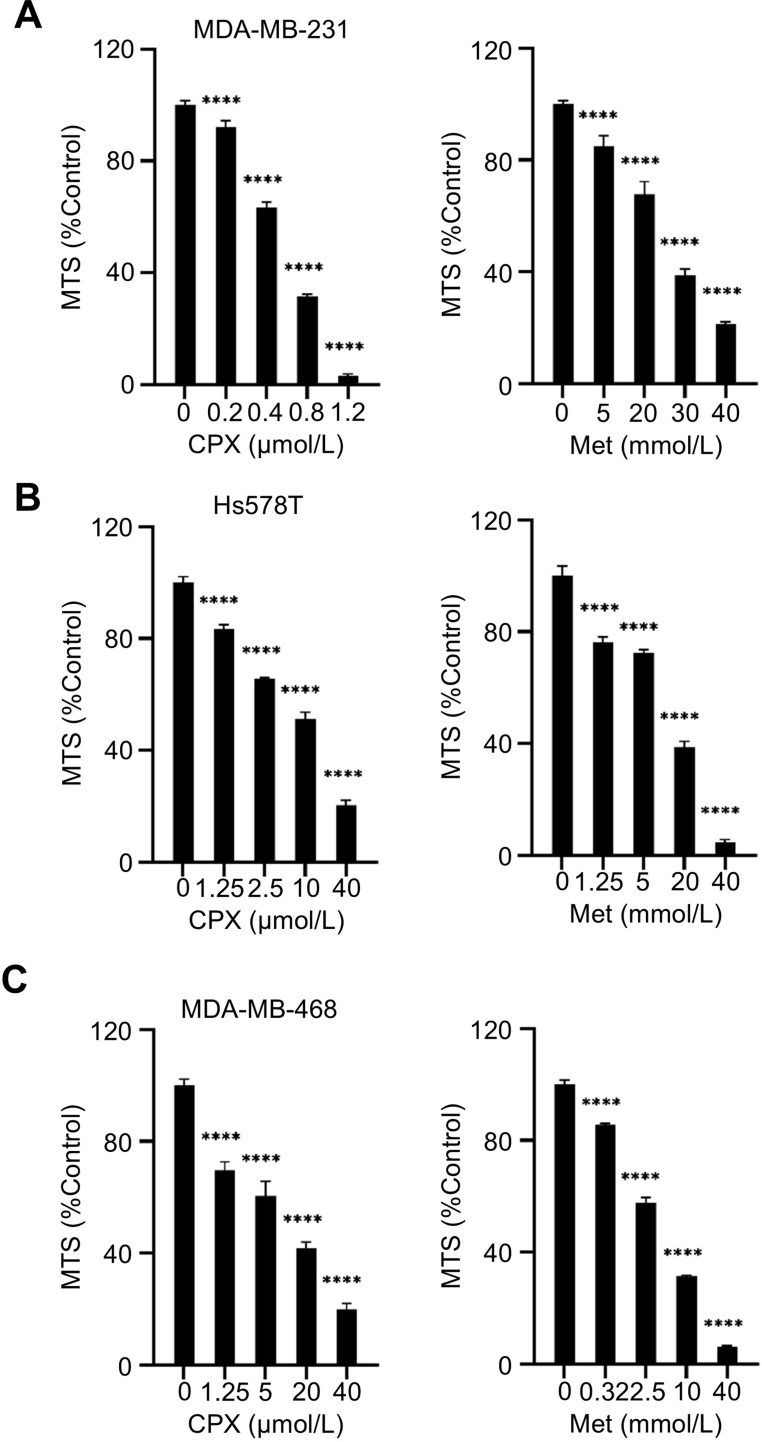


**figs2:**
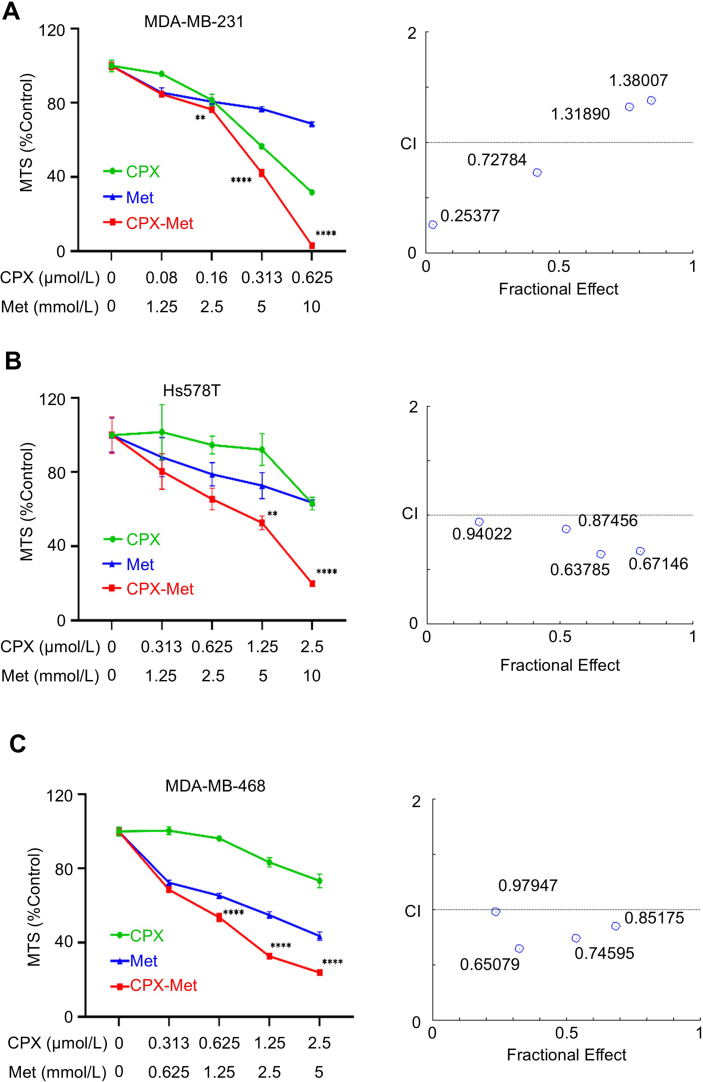


**figs3:**
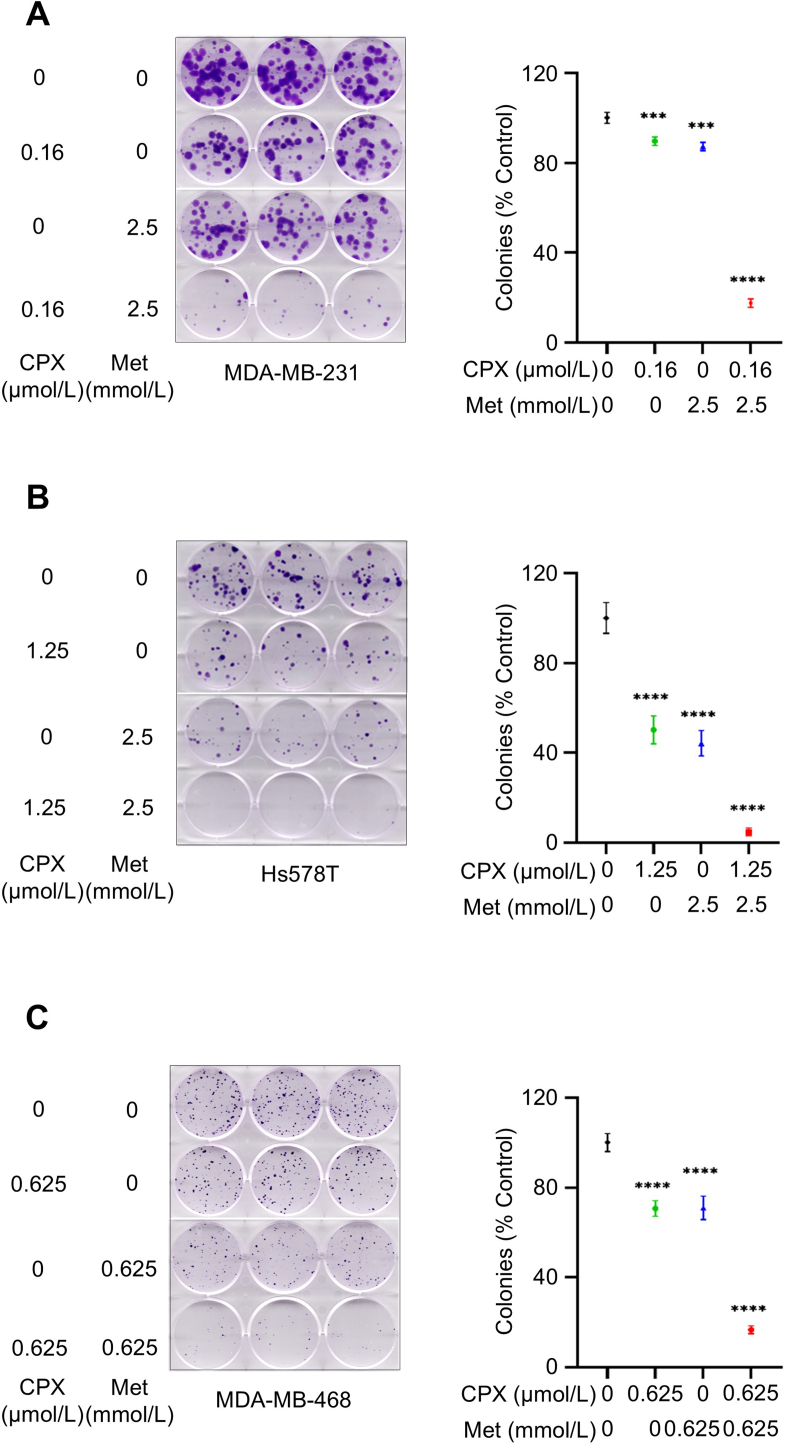


**figs4:**
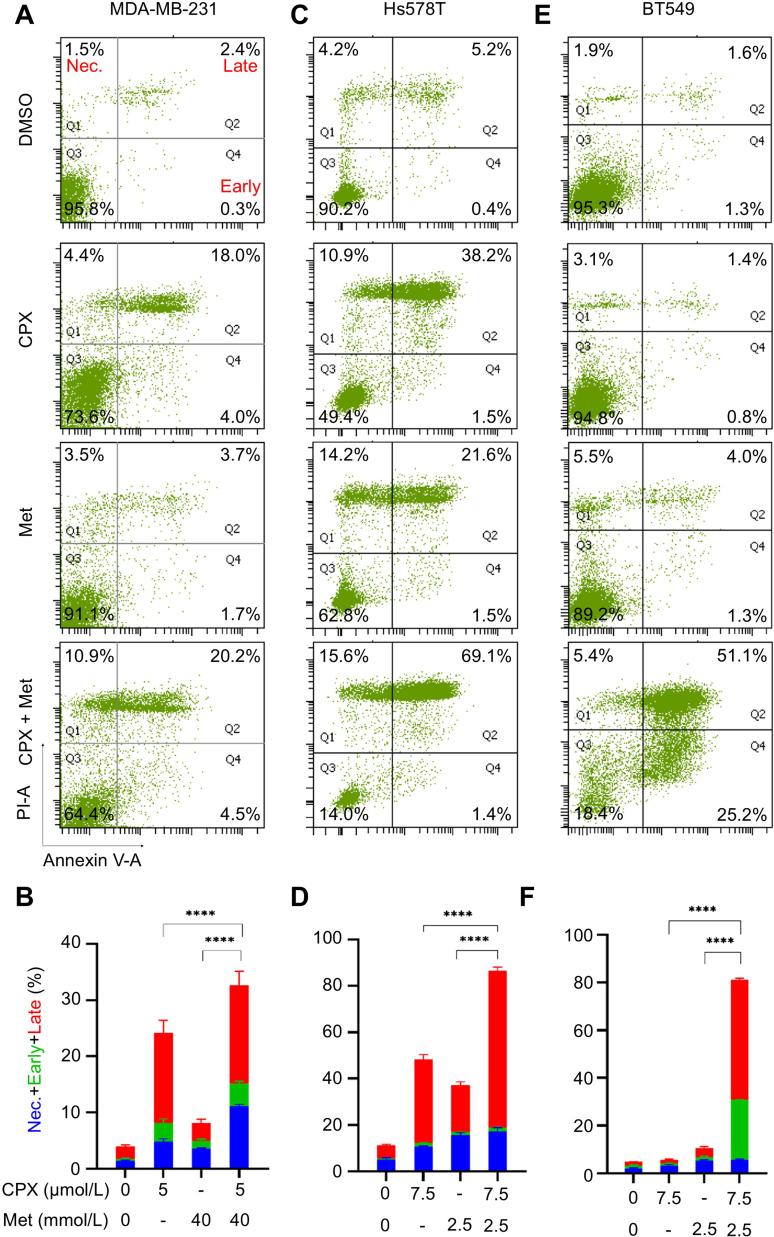


**figs5:**
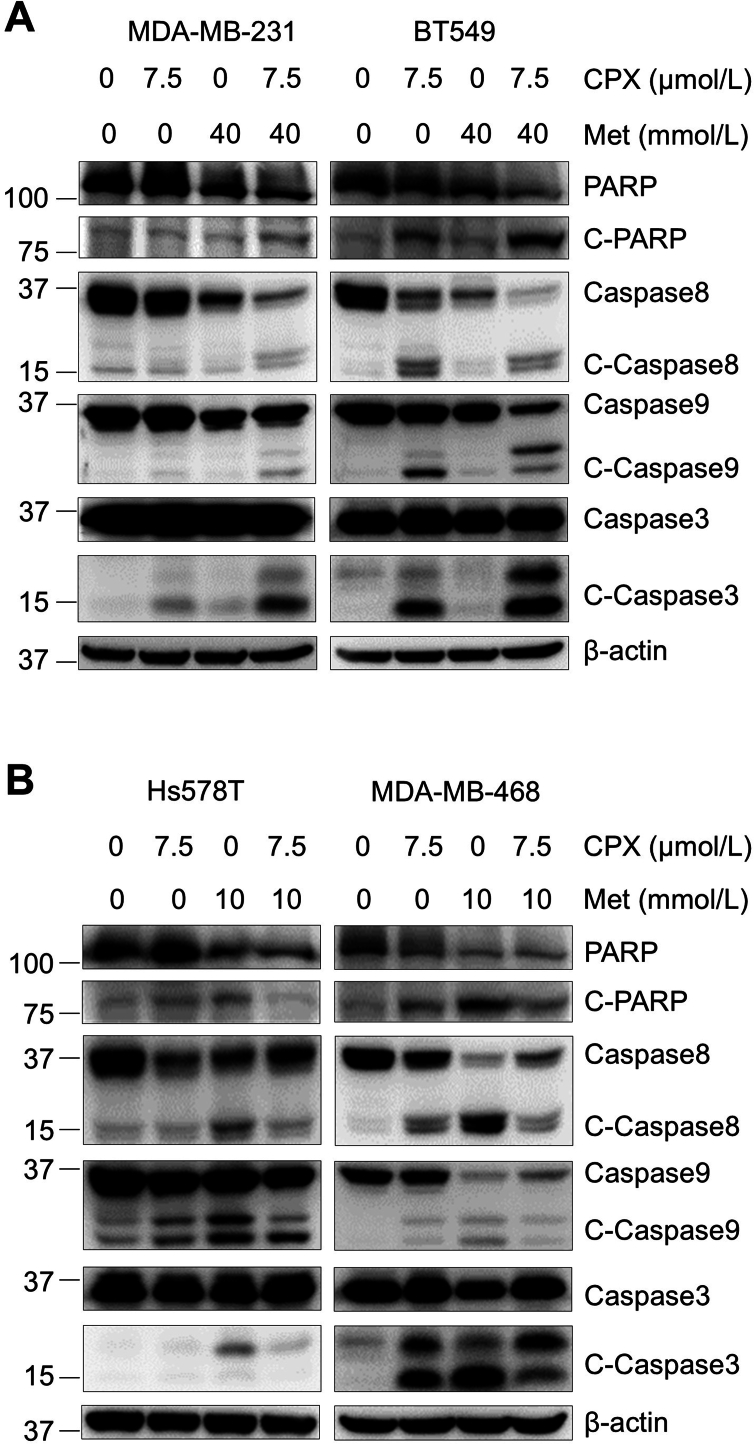


**figs6:**
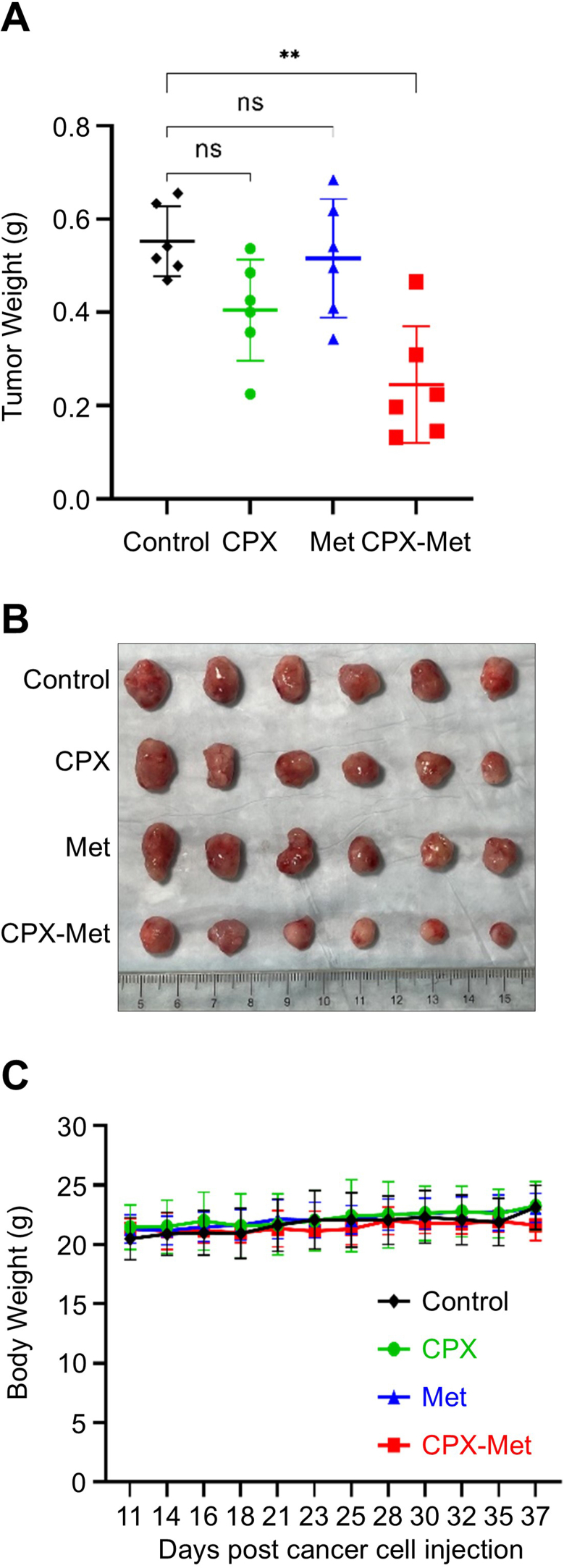


## References

[1] Liu Y., Hu Y., Xue J. (2023). Advances in immunotherapy for triple-negative breast cancer. Mol Cancer.

[2] Mohi-Ud-Din R., Chawla A., Sharma P. (2023). Repurposing approved non-oncology drugs for cancer therapy: a comprehensive review of mechanisms, efficacy, and clinical prospects. Eur J Med Res.

[3] Cheng T., Wang C., Lu Q. (2022). Metformin inhibits the tumor-promoting effect of low-dose resveratrol, and enhances the anti-tumor activity of high-dose resveratrol by increasing its reducibility in triple negative breast cancer. Free Radic Biol Med.

[4] Wan X., Xiang J., Fan H. (2023). Ciclopirox olamine induces proliferation inhibition and protective autophagy in hepatocellular carcinoma. Pharmaceuticals (Basel).

[5] Song J., Du J., Han L., Lin X., Fan C., Chen G. (2023). The effect of metformin on triple-negative breast cancer cells and nude mice. Altern Ther Health Med.

