# Supplementary material for: Synergistic anti-tumor activity of ciclopirox olamine and metformin in triple-negative breast cancer
Source: Genes Dis. 2025 Jan 22;13(2):101538. doi: 10.1016/j.gendis.2025.101538 (PMC12596565; doi:10.1016/j.gendis.2025.101538)
Supplement: Multimedia component 1 [file mmc1.docx]

**Supplemental information**

**Materials and Methods**

**Reagents and antibodies**

Metformin Hydrochloride was purchased from Tokyo Chemical Industry Co., Ltd. (TCI). One Molar stock solution was prepared by dissolving the powder in sterile water. Ciclopirox Olamine (sc-204688A) was purchased from Santa Cruz Biotechnology Inc. and dissolved in DMSO to make a 20 mM stock solution. The reagents were stored at -20°C for future use. Antibodies β-actin (8H10D10), caspase-8 (1C12), caspase-9, caspase-3 (8G10), cleaved caspase-3 (Asp175), Ki-67 (D2H10), PARP (46D11), were purchased from Cell Signaling Technology, Inc. Apoptosis detection kit (640932) was purchased from BioLegend.

**Cells and cell culture**

Human TNBC cell lines (HCC1806, MDA-MB-231, MDA-MB-468, BT549, and Hs578T) were maintained in DMEM/F12 supplemented with 5% or 10% fetal bovine serum (FBS) and cultured at 37°C, 5% CO_2_. For experimental treatments, cells were seeded in 96-well plates, 6-well plates, or 6-cm dishes, depending on the assay being conducted.

**Cell proliferation assay**

Cell viability was assessed using the MTS assay (Promega, USA) as described previously ^1-3^. Briefly, cells were seeded onto 96-well plates with medium containing 10% FBS. Twenty-four hours later, the culture medium was replaced with fresh medium containing 5% FBS (control) or the same medium containing different concentrations of CPX, Met, or CPX plus Met. The cells were cultured for another 72 hours. After treatment, the MTS reagent was added and incubated for an additional 1-3 hours at 37°C. Absorbance was measured using a Synergy LX Multi-Mode Reader (Biotek, Winooski, VT, USA) to determine the viable cells relative to the control group, defined as 100% survival.

**Colony formation assay**

As described previously ^2^, cells at the exponential growth phase were harvested, seeded in 6-well plates, and allowed to adhere for 24 hours. Post-adherence, the medium was replaced with fresh medium containing different concentrations of CPX, Met, or CPX plus Met, respectively. The culture medium was changed every third day. After two weeks, colonies were stained, images were captured using a digital scanner, and quantified using the Image J software.

**Flow cytometry**

Cell apoptosis was assessed through flow cytometry analysis ^2;4^. Briefly, cells cultured in 6-well plates were treated with fresh medium containing the respective reagents and cultured for 48 hours. All cells were collected and cell apoptosis was analyzed using BD FACSymphony™ A3. Data were processed using the FlowJo software.

**Western blot analysis**

After treatments, cells were lysed, and equal amounts of total cell lysates were subjected to western blot assays. The blots were probed with antibodies specific to PARP, caspase-8, caspase-9, caspase-3, and β-actin. Signals were detected using the Pierce ECL Western Blotting Substrate from Thermo Scientific.

**Tumor xenograft model**

Athymic nu/nu mice were maintained following the Institutional Animal Care and Use Committee (IACUC) procedures and guidelines. HCC1806 cells (1 × 10^6^) were suspended in 100 μL of PBS, mixed with 50% Matrigel (BD Biosciences), and injected subcutaneously into the fat pads of six-week-old female athymic mice. Tumor volume was measured five times weekly, and mouse body weight thrice weekly. The tumor volume was calculated by the formula: Volume = (Length × Width^2^)/2. When tumors reached ~60mm^3^, mice were randomly assigned into four groups (n=6) and treated daily with solution control, CPX (20 mg/kg), Met (350 mg/kg), or CPX plus Met by oral gavage. The tumor growth curves were plotted using average tumor volume and followed by statistical analysis as we described previously ^5-8^. At the end of treatment, mice were euthanized according to the approved IACUC protocol. Tumors were dissected, imaged, and measured for weight. Samples from all animals were fixed and embedded in paraffin for immunohistochemical analyses.

**Immunohistochemistry (IHC) assay**

IHC assays were performed as we previously described ^1;9^. In brief, five-micron-thick paraffin sections were deparaffinized, antigen retrieved, and immunohistochemically stained for Ki67 and cleaved caspase-3 overnight (4 °C). After washing with PBS, the slides were incubated with Streptavidin-HRP for 30 min, followed by twice washing with PBS. The staining colors were developed with DAB and counterstaining with hematoxylin. Finally, all sections were mounted with a permanent aqueous mounting medium (Bio-Rad).

**Quantification of IHC analysis**

As reported previously^10^, ImageJ was used to quantify the IHC staining analysis. After being imported into the software, images were changed to an 8-bit grayscale type, and the threshold was adjusted to show the DAB-stained areas. The mean intensity was measured using ImageJ's "Measure" function. Five fields of each group were assessed.

**Statistical analysis**

Data were expressed as mean ± standard deviation. Differences between groups were evaluated using two-way ANOVA analysis to determine significance. A *p*-value of less than 0.05 was considered statistically significant. All statistical analyses were performed using GraphPad Prism 9 software.

**Supplementary Figures**

**
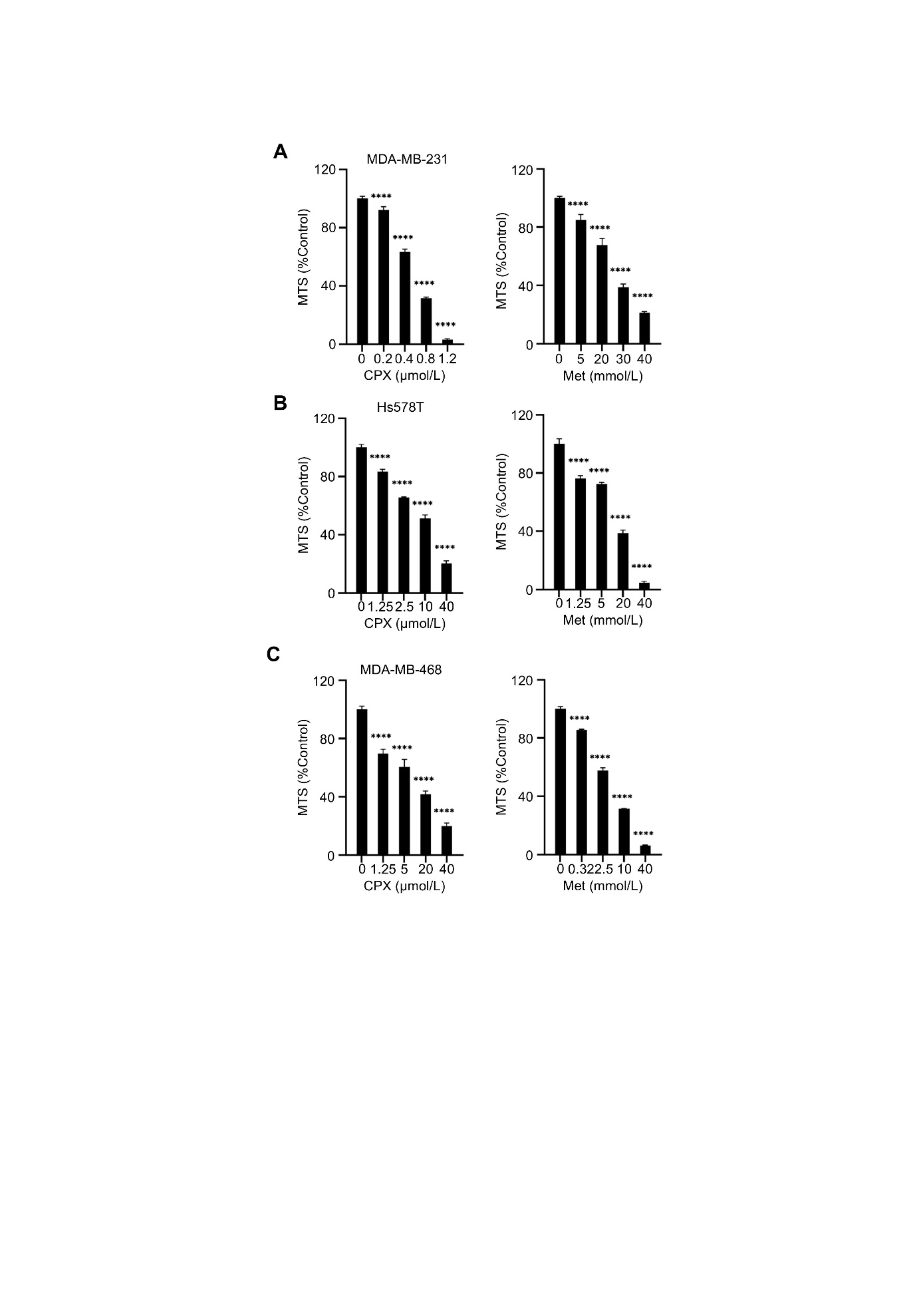
**

**Figure S1** Both CPX and Met inhibited the proliferation of TNBC cells in a dose-dependent manner. TNBC cells (MDA-MB-231 **(A)**, Hs578T **(B)**, and MDA-MB-468 **(C)**) were plated into 96-well plates with 0.1 mL DMEM/F12 (1:1) medium supplemented with 10% fetal bovine serum (FBS). Cells were treated with fresh medium containing 5% FBS (control) or the same medium containing indicated concentrations of CPX (left panels) or Met (right panels) and incubated for another 72 h. After incubation, the percentages of surviving cells from each cell line relative to controls, defined as 100% survival, were determined by MTS assays. (^✱✱✱✱^*P*<0.0001). Bars: SD.


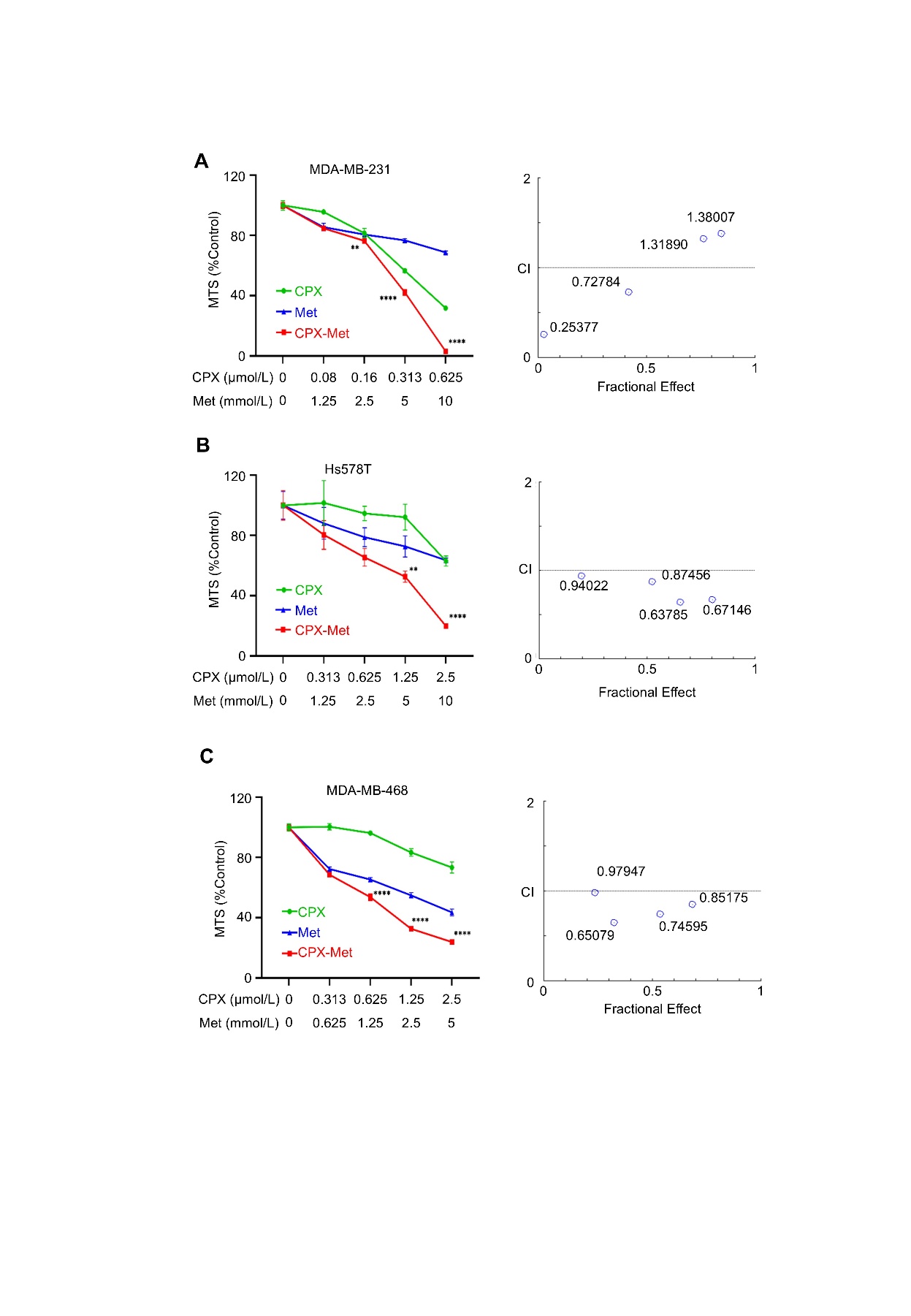


**Figure S2** Combinations of CPX and Met exhibited synergistic effects that inhibited the proliferation and survival of TNBC cells. TNBC cells (MDA-MB-231 **(A)**, Hs578T **(B)**, and MDA-MB-468 **(C)**) were plated into 96-well plates with 0.1 mL DMEM/F12 (1:1) medium supplemented with 10% fetal bovine serum (FBS). Cells were treated with fresh medium containing 5% FBS (control) or the same medium containing indicated concentrations of CPX, Met, or CPX plus Met. 72 h later, the percentages of surviving cells (left panels) from each cell line relative to controls, defined as 100% survival, were determined by MTS assays. The combination index (right panels) of CPX and Met in the treatment of TNBC cells (MDA-MB-231 **(A)**, Hs578T **(B)**, and MDA-MB-468 **(C)**) was calculated with the Chou-Talalay method. (^✱✱^*P*<0.01, ^✱✱✱✱^*P*<0.0001). Bars: SD.

**
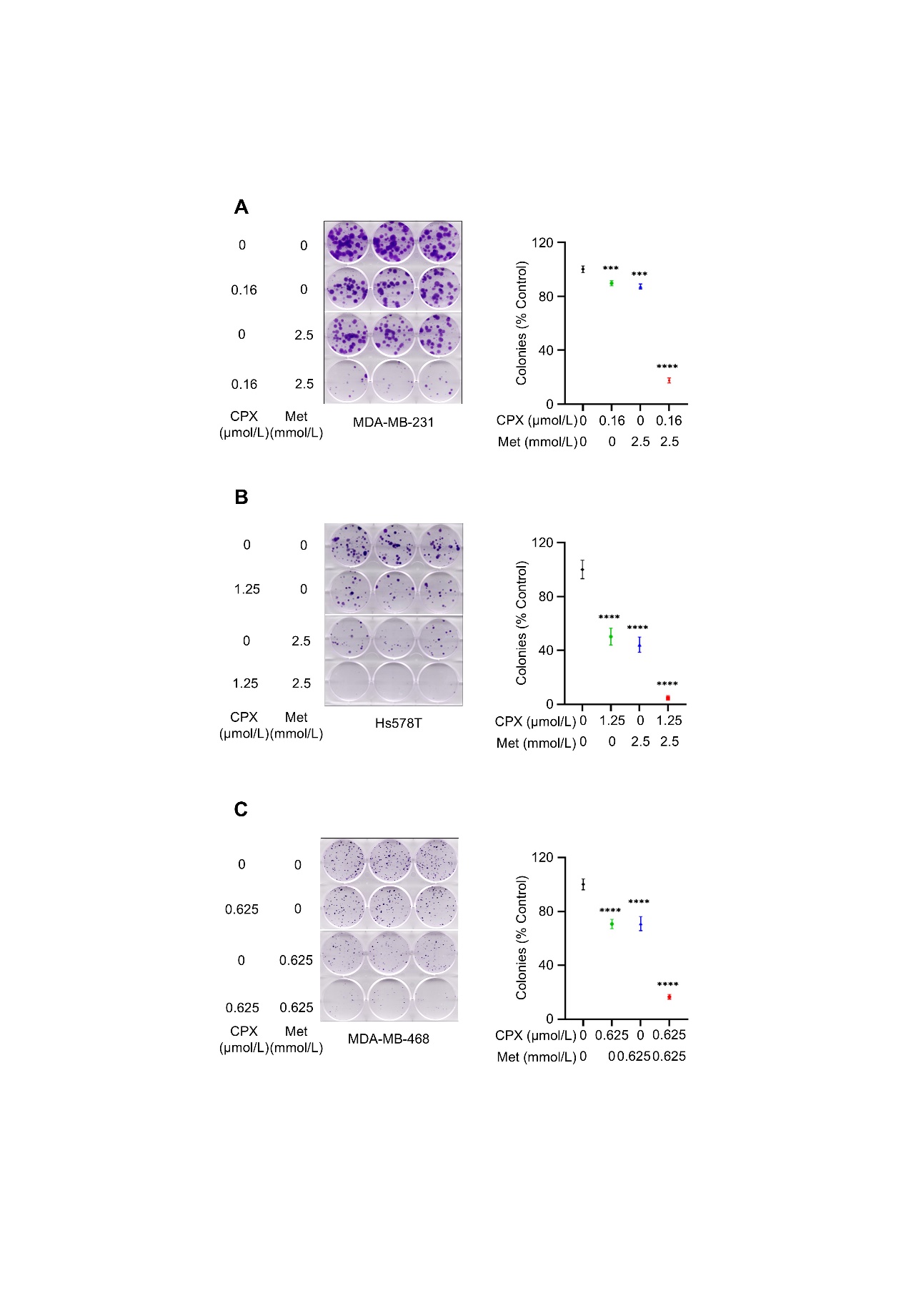
**

**Figure S3** CPX in combination with Met markedly suppressed colony formation of TNBC cells. TNBC cells (MDA-MB-231 **(A)**, Hs578T **(B)**, and MDA-MB-468 **(C)**) were plated into 6-well plates in triplicate in 2 mL of medium containing 10% FBS. Cells were treated with fresh medium containing 5% FBS (control) or the same medium containing different concentrations of CPX, Met, or CPX plus Met as indicated. The culture medium was then changed every three days for two weeks. Representative images (left panels) of the clonogenic assay for each cell line were taken by a digital scanner, and the relevant quantification of the number of colonies (right panels) was performed by calculating the percentages of colony numbers from each cell line relative to controls, defined as 100%, measured by Image J. (^✱✱✱^*P*<0.001, ^✱✱✱✱^*P*<0.0001). Bars: SD.

**
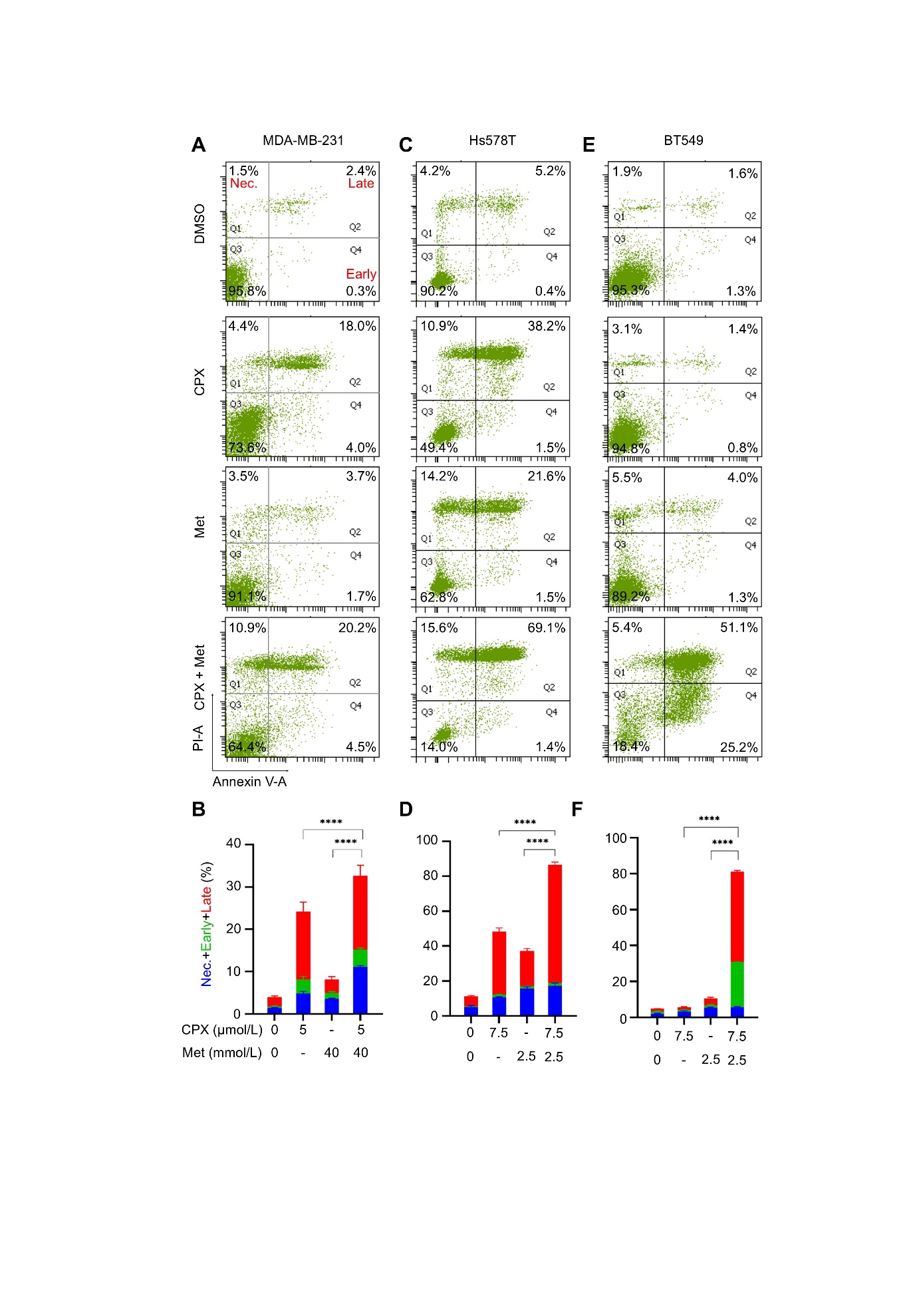
**

**Figure S4** CPX combined with Met profoundly induced cell death in TNBC cells. TNBC cells (MDA-MB-231, Hs578T, and MDA-MB-468) were cultured in 6 cm dishes with medium containing 10% FBS. After 24 h, cells were treated with indicated concentrations of CPX, Met, or CPX plus Met in a fresh medium containing 5% FBS for another 48 h. All cells were collected and analyzed by flow cytometry; representative data were shown in **(A, C, E)**. The relevant quantification analysis was performed by calculating the percentages of the Necrosis (Nec.), early apoptosis (Early), and late apoptosis (Late) population of cells from three independent replicates of the experiments **(B, D, F)**. (^✱✱✱✱^*P*<0.0001). Bars: SD.

**
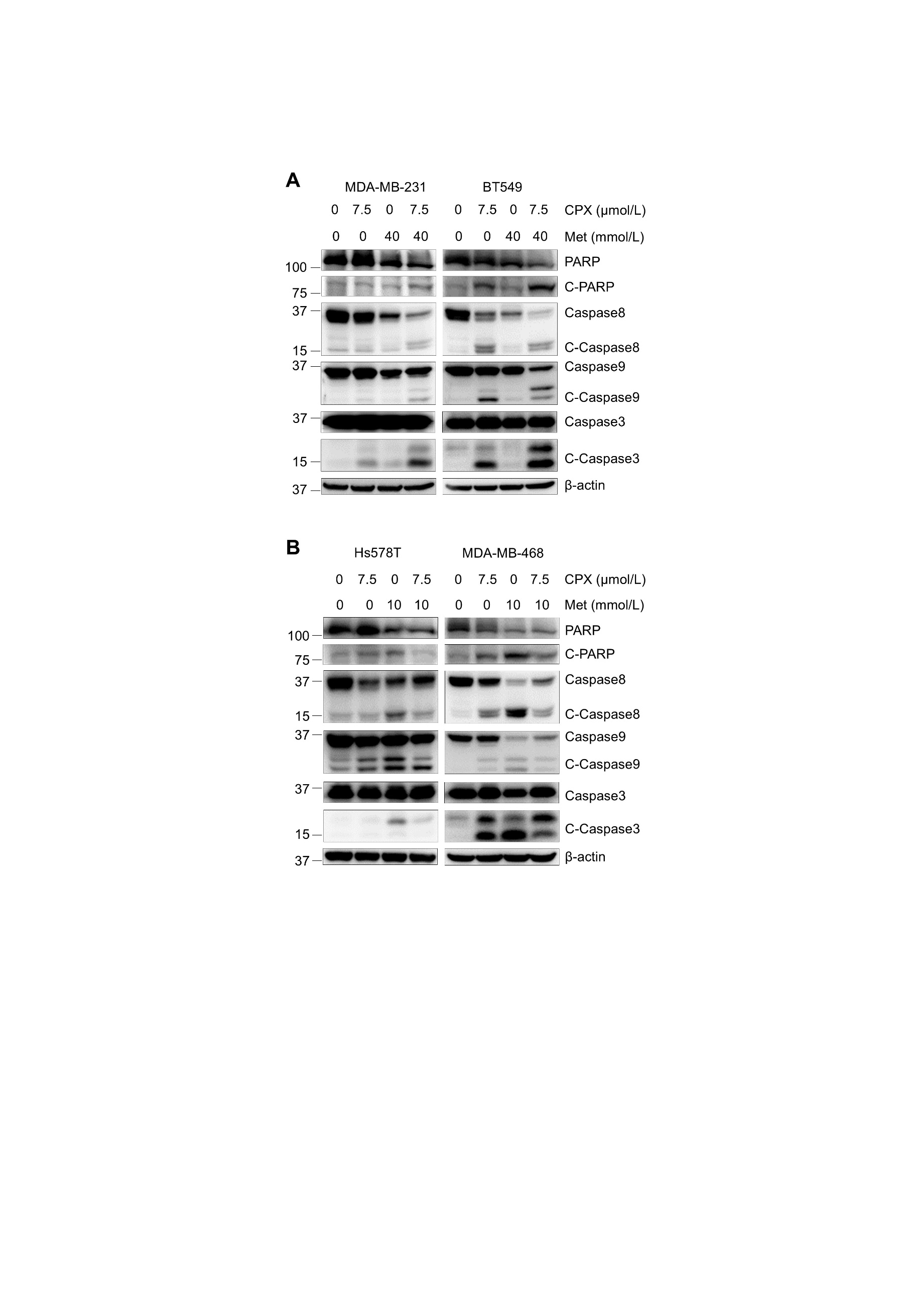
**

**Figure S5** CPX and Met together enhanced PARP cleavage and activation of caspases in TNBC cells. TNBC cells (MDA-MB-231 and BT549 **(A)**, Hs578T and MDA-MB-468 **(B)**) were treated with indicated concentrations of CPX, Met, or CPX plus Met in a fresh medium containing 5% FBS and incubated for 48 h. All cells were collected, and the cell lysates were used for western blot analyses with specific antibodies against PARP, caspase-8, caspase-9, caspase-3, and β-actin, as indicated.

**
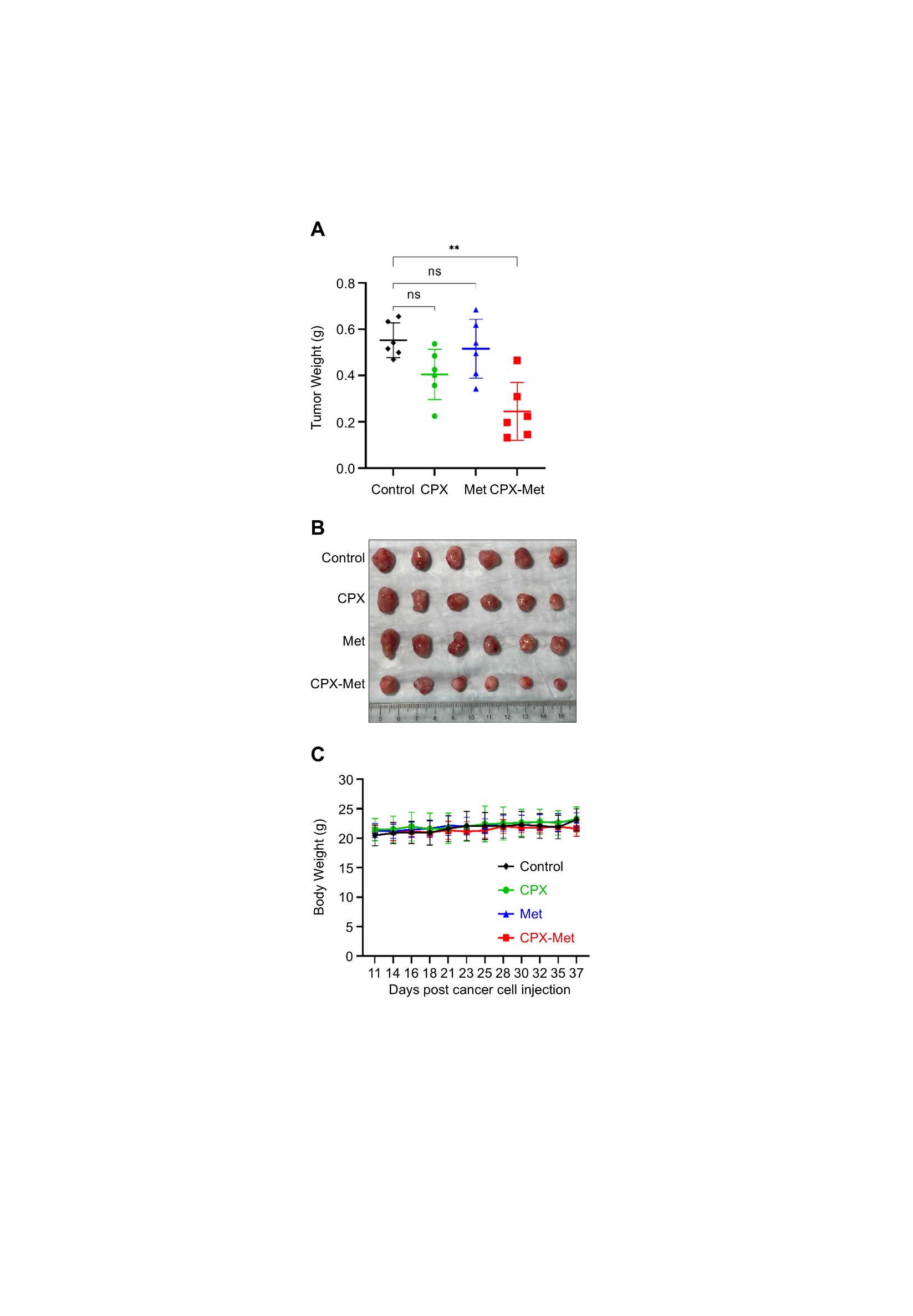
**

**Figure S6** Combinations of CPX and Met enhanced *in vivo* antitumor effects. At the end of treatment, tumor-bearing mice from the control and drug-treated groups were sacrificed. The tumors were dissected, measured for weight **(A),** and imaged as indicated **(B)**. **(C)** Mouse body weight during the treatment was shown. Bars: SD. (***P* < 0.01), Bars: SD.

References

1. Jingcao Huang SW, Hui Lyu, Bo Cai, XiaoHe Yang, Jianxiang Wang and Bolin Liu. The anti-erbB3 antibody MM-121SAR256212 in combination with trastuzumab exerts potent antitumor activity against trastuzumab-resistant breast cancer cells. *Molecular Cancer.* 2013;12(134).

2. Hui Lyu XHY, Susan M. Edgerton, Ann D. Thor, Xiaoying Wu, Zhimin He, and Bolin Liu. The erbB3- and IGF-1 receptor-initiated signaling pathways exhibit distinct effects on lapatinib sensitivity against trastuzumab-resistant breast cancer cells. *Oncotarget.* 2016;7(3):2921-2935.

3. Liu S, Polsdofer EV, Zhou L, et al. Upregulation of endogenous TRAIL-elicited apoptosis is essential for metformin-mediated antitumor activity against TNBC and NSCLC. *Mol Ther Oncolytics.* 2021;21:303-314.

4. Lyu H, Hou D, Liu H, et al. HER3 targeting augments the efficacy of panobinostat in claudin-low triple-negative breast cancer cells. *NPJ Precis Oncol.* 2023;7(1):72.

5. Huang J, Wang S, Lyu H, et al. The anti-erbB3 antibody MM-121/SAR256212 in combination with trastuzumab exerts potent antitumor activity against trastuzumab-resistant breast cancer cells. *Molecular Cancer.* 2013;12(1):134.

6. Wang S, Huang J, Lyu H, et al. Therapeutic targeting of erbB3 with MM-121/SAR256212 enhances antitumor activity of paclitaxel against erbB2-overexpressing breast cancer. *Breast cancer research.* 2013;15(5):R101.

7. Wang S, Zhu L, Zuo W, et al. MicroRNA-mediated epigenetic targeting of Survivin significantly enhances the antitumor activity of paclitaxel against non-small cell lung cancer. *Oncotarget.* 2016;7(25):37693-37713.

8. Liu H, Ruan S, Larsen ME, Tan C, Liu B, Lyu H. Trastuzumab-resistant breast cancer cells-derived tumor xenograft models exhibit distinct sensitivity to lapatinib treatment in vivo. *Biol Proced Online.* 2023;25(1):19.

9. Lyu H, Shen F, Ruan S, et al. HER3 functions as an effective therapeutic target in triple negative breast cancer to potentiate the antitumor activity of gefitinib and paclitaxel. *Cancer Cell Int.* 2023;23(1):204.

10. Zheng J, Zhao S, Yu X, Huang S, Liu HY. Simultaneous targeting of CD44 and EpCAM with a bispecific aptamer effectively inhibits intraperitoneal ovarian cancer growth. *Theranostics.* 2017;7(5):1373-1388.
